# Modulation of therapy-induced senescence by reactive lipid aldehydes

**DOI:** 10.1038/cddiscovery.2016.45

**Published:** 2016-07-04

**Authors:** A C Flor, A P Doshi, S J Kron

**Affiliations:** 1 Ludwig Center for Metastasis Research, Department of Molecular Genetics and Cell Biology, The University of Chicago, Chicago, IL 60637, USA

## Abstract

Current understanding points to unrepairable chromosomal damage as the critical determinant of accelerated senescence in cancer cells treated with radiation or chemotherapy. Nonetheless, the potent senescence inducer etoposide not only targets topoisomerase II to induce DNA damage but also produces abundant free radicals, increasing cellular reactive oxygen species (ROS). Toward examining roles for DNA damage and oxidative stress in therapy-induced senescence, we developed a quantitative flow cytometric senescence assay and screened 36 redox-active agents as enhancers of an otherwise ineffective dose of radiation. While senescence failed to correlate with total ROS, the radiation enhancers, etoposide and the other effective topoisomerase inhibitors each produced high levels of lipid peroxidation. The reactive aldehyde 4-hydroxy-2-nonenal, a lipid peroxidation end product, was sufficient to induce senescence in irradiated cells. In turn, sequestering aldehydes with hydralazine blocked effects of etoposide and other senescence inducers. These results suggest that lipid peroxidation potentiates DNA damage from radiation and chemotherapy to drive therapy-induced senescence.

## Introduction

Accelerated senescence (AS) is considered a form of premature cellular aging characterized by irreversible proliferative arrest accompanied by characteristic changes in gene expression, metabolism and cell morphology. AS is indistinguishable from replicative senescence (RS), except that onset of senescence is independent of telomere integrity. Instead, onset of AS has been ascribed to diverse cellular insults such as oncogene activation, chromatin disruption, unrepairable chromosomal damage and oxidative stress.^1–3^

Even though cancer cells resist RS due to re-expression of telomerase, significant levels of unrepairable DNA damage can successfully induce AS in these cells.^4^ Laboratory and clinical evidence show that conventional cancer treatments including chemotherapy and radiation induce AS in tumors,^5,6^ a process termed therapy-induced senescence (TIS). Untangling the pathways to senescence in cancer cells has been challenging, as increased reactive oxygen species (ROS) and DNA damage are shared outcomes of exposure to common therapies.^7,8^ Although considerable uncertainty remains whether TIS is a desirable outcome of cancer treatment,^9–11^ recent studies suggest that senescent cells in tumors may have beneficial effects, including stimulation of antitumor immunity. As such, we and others have sought new chemical probes that can dissect determinants of cancer cell senescence *in vitro* and that may modulate senescence *in vivo* toward investigating impact on efficacy of chemotherapy and radiation treatment.

To date, few successful chemical screens have been completed to detect small-molecule modulators of senescence.^12^ While senescent cells display a wide range of morphological and biochemical features that may distinguish them from proliferating cells,^13^ most studies have relied solely on detection of senescence-associated *β*-galactosidase (SA-*β*-Gal), despite its significant weaknesses and caveats.^14–16^ The conventional SA-*β*-Gal assay^17^ is based on imaging blue staining by the chromogenic substrate X-Gal (5-bromo-4-chloro-3-indolyl-*β*-d-galactopyranoside) in fixed and permeabilized cells, making it poorly suited as a screening assay. The fluorescein galactoside C_12_-FDG (5-dodecanoyl-aminofluorescein-di-*β*-d-galactopyranoside) can detect SA-*β*-Gal in living cells,^18^ but the green fluorescent signal overlaps autofluorescence (AF) from the age-related pigment lipofuscin.^19^

Toward establishing a high-throughput, live-cell screening tool, we developed a novel multiparameter flow cytometry assay for senescence based on tracking cell size and viability, detecting SA-*β*-Gal by cleavage of the near-infrared *β*-galactosidase reporter DDAOG (9*H*-(1,3-dichloro-9,9-dimethylacridin-2-one-7-yl)-*β*-D-galactopyranoside), measuring accumulation of lipofuscin as green AF and detecting ROS with a violet probe.^20^ We applied the screen to evaluate a panel of 36 redox-modulating compounds, with and without a single, sensitizing dose of radiation, for their ability to elevate ROS and promote senescence in two murine melanoma cell lines.

Although ROS appeared to have a poor correlation with senescence overall, we discovered a new connection to lipid peroxidation (LPO). Investigating the strong senescence-inducing effect of the redox-active topoisomerase II inhibitor etoposide (VP-16) revealed high levels of cellular LPO. The effects of etoposide were recapitulated by treating cells with both radiation and aldehyde end products of LPO. In turn, the aldehyde-scavenging compound hydralazine HCl (HYD)^21,22^ prevented senescence induction by etoposide and other topoisomerase inhibitors, including camptothecin and doxorubicin. These findings provide insight into a new mechanism underlying cellular senescence, and may provide a mechanistic basis by which etoposide and other agents can efficiently promote TIS.

## Results

### Quantitative measurement of *γ-*irradiation-induced senescence and ROS

Toward enhancing detection of SA-*β*-Gal by flow cytometry and avoiding cross-talk with cellular AF, we evaluated the cell-permeable *β*-galactosidase probe DDAOG.^23,24^ DDAOG is itself fluorescent, but hydrolysis to release DDAO (7-hydroxy-9*H*-(1,3-dichloro-9,9-dimethylacridin-2-one)) shifts peak excitation >200 nm and emission 50 nm, from 610 (orange) to 660 nm (near infrared). Compared with detection of SA-*β*-Gal with X-Gal or C_12_-FDG by microscopy, the DDAO assay displayed comparable sensitivity and specificity (Supplementary Figure S1). Applied to flow cytometry, the near-infrared channel on commercial flow cytometers readily detects DDAO but is insensitive to AF, enabling independent detection of SA-*β*-Gal and lipofuscin in separate channels. Thus, we developed a flow cytometry assay in which we evaluated SA-*β*-Gal at 660 nm and AF at 525 nm to quantify the level of senescence in irradiated cells, while also following the fluorescent ROS probe Calcein Violet at 450 nm (CV450).

**Figure 1:**
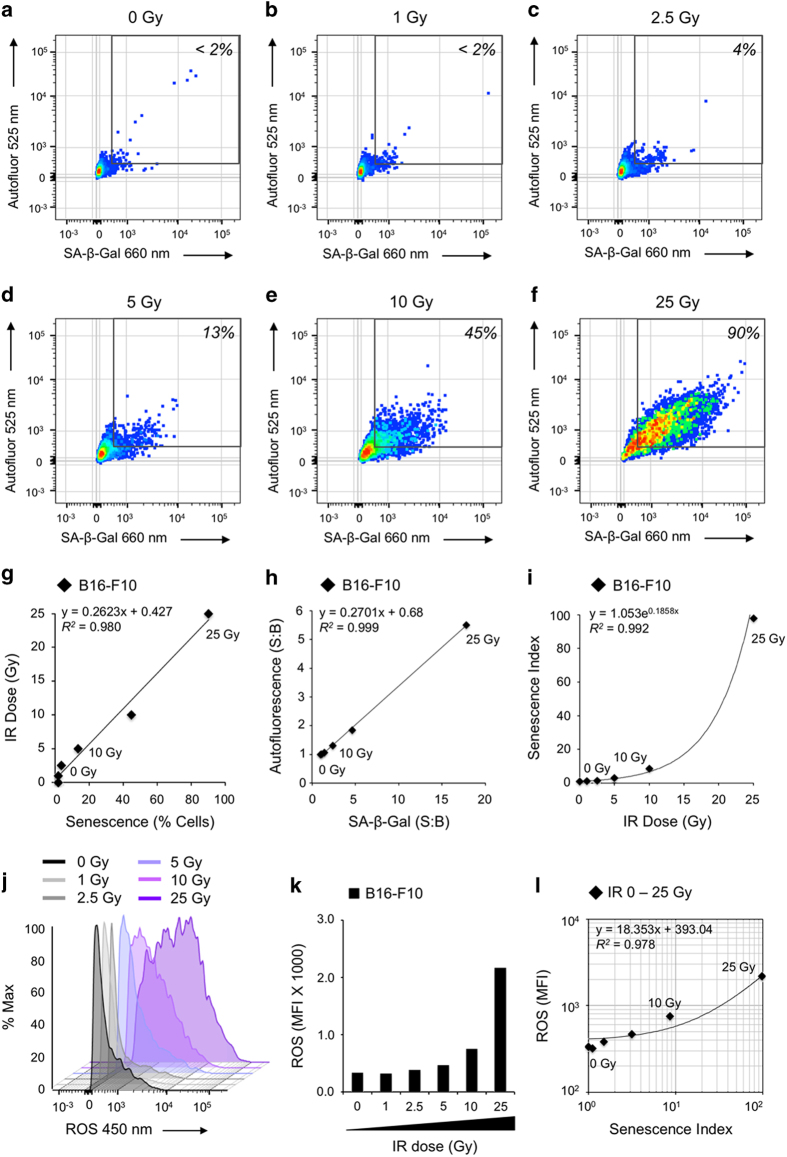
Quantitative measurement of IR-induced senescence and ROS. (**a**–**f**) Flow cytometric dot plots indicate increasing senescence with IR dose evaluated by independently measuring SA-*β*-Gal and AF in each cell. (**g**) Plotting percent senescent cells of total cell population *versus* IR dose (0–25 Gy) reveals linear correlation. (**h**) Linear correlation of SA-*β*-Gal and AF for cell populations exposed to increasing IR doses (0–25 Gy). (**i**) Exponentially increasing relationship of IR dose (Gy) *versus* SI. SI is a novel metric for senescence derived from multiplying the median values for SA-*β*-Gal and AF. (**j**) Flow cytometry histograms showing ROS induced by increasing IR dose. (**k**) Median fluorescence intensity (MFI) of ROS data for increasing IR dose as shown in (**j**). (**l**) SI *versus* ROS (MFI). Data are shown on log/log scale with linear regression plotted (*R*^2^=0.98).

To validate the multiparameter senescence assay, we subjected B16-F10 cells to increasing doses of ionizing irradiation (IR) and analyzed cells for SA-*β*-Gal at 660 nm and AF at 525 nm. Ten thousand cells were analyzed per condition. Dead cells and debris were excluded from the analysis using a viability stain. A gate was set to identify senescent cells such that <2% of unirradiated (0 Gy) cells were included within the gate (Figure 1a). The percent of cells falling in the senescent cell gate increased over the range from 0 to 25 Gy, reaching nearly 100% of viable cells (Figures 1b–f). Plotting these data indicated that the percentage of senescent cells was proportional to IR dose (Figure 1g; *R*^2^=0.980). We next calculated signal to background (S:B) for median SA-*β*-Gal and AF and plotted the data, which revealed a linear correlation with IR dose (Figure 1h; *R*^2^=0.999). Toward defining a score to facilitate comparison among senescence-inducing conditions, we multiplied SA-*β*-Gal and AF (S:B) to yield a senescence index (SI) that displayed an exponential increase with IR dose (Figure 1i; *R*^2^=0.992).

### Flow cytometric screen to quantify senescence induction by redox-modulating compounds combined with ionizing radiation

Examination of the irradiated B16-F10 cells with CV450 (Figures 1j and k) revealed a dose-dependent increase in ROS from 0 to 25 Gy, much like SA-*β*-Gal and AF. Plotting SI *versus* ROS revealed a proportional relationship (Figure 1l; *R*^2^=0.978). This raised the questions whether increased ROS was an intrinsic feature of senescent cells and would simply increasing ROS be sufficient to enhance senescence.

Using the dual parameter cytometric cell screening assay, we evaluated 36 redox-modulating compounds for their ability to modulate DNA damage-induced senescence. The panel of redox-modulating compounds included oxidizers and antioxidants, as well as drugs affecting redox-related cellular functions and pathways. We included a second B16 murine melanoma cell line, B16-F1, which exhibits distinct endogenous ROS levels and antioxidant potential compared with B16-F10. Further, the F1 variant is radiosensitive, whereas the F10 variant is radioresistant. To sensitize cells to the effects of ROS on senescence, we treated them with 5 Gy, a dose sufficient to induce ~10% senescence in each cell line (Figure 1d and Supplementary Figure S2).

**Figure 2:**
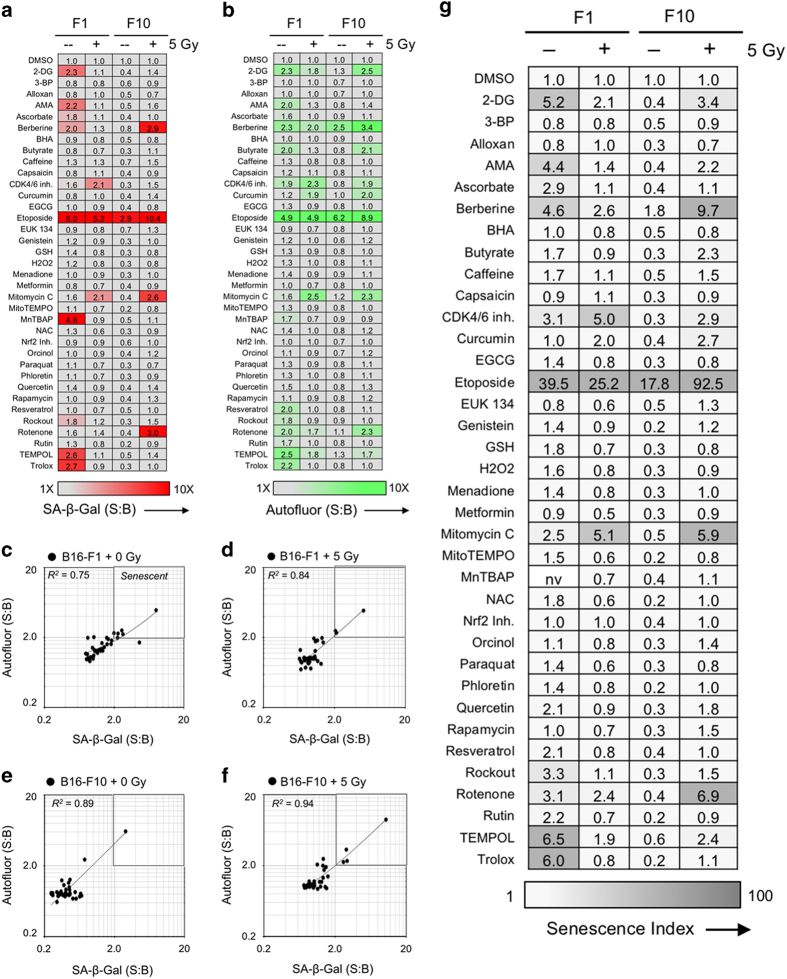
Flow cytometric senescence screen of redox-modulating compounds±low-dose IR. (**a** and **b**) Heat maps showing screening results for 36 known redox-modulating compounds added to B16 melanoma cell line variants F1 and F10. Cells were subjected to either 0 or 5 Gy γ-IR and screened in 96-well plates. (**a**) SA-*β*-Gal was measured at 660 nm using near-infrared probe DDAO-Gal and (**b**) cellular AF was measured at 525 nm using a stain-free approach. Signal *versus* background (S:B) data as shown were calculated as average MFI of duplicate experimental samples divided by untreated control. (**c**–**f**) Statistical analysis of correlation (*R*^2^) between SA-*β*-Gal and cellular AF for identification of senescence-inducing conditions. Data points were taken from screening analysis shown in (**a** and **b**). Etoposide is the outlier in each data plot. Results indicate that across cell lines and conditions, SA-*β*-Gal and AF were generally correlated. Conditions that exhibited ≥2.0-fold elevation for both SA-*β*-Gal and AF were considered screening hits. Values for SA-*β*-Gal and AF are multiplied to determine the SI as a single metric measuring senescence induction for each condition. (**g**) Heat map showing SI values for screening conditions. The single metric allows at-a-glance interpretation of senescence screening data obtained rapidly using living cells.

**Figure 3:**
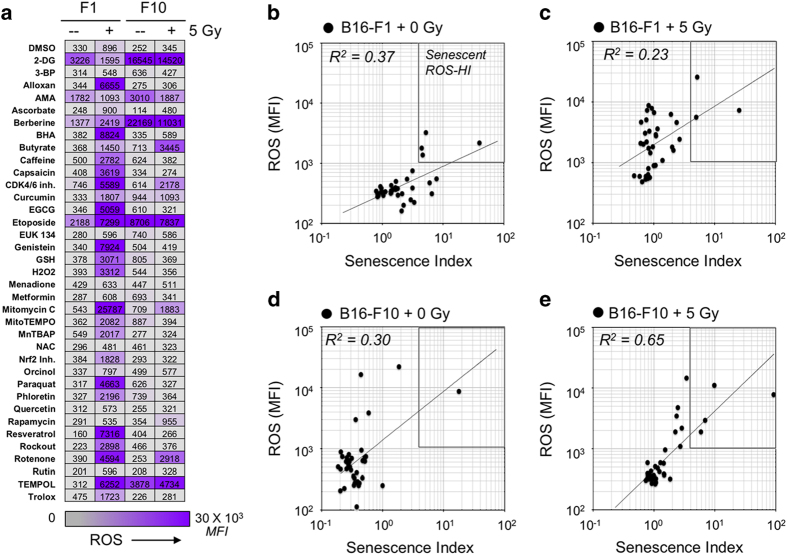
Flow cytometric ROS screening results. (**a**) During the senescence screening assay presented in Figure 2, ROS was concurrently measured at 450 nm; data shown were calculated as average median fluorescence intensity (MFI) of duplicate experimental samples. Results demonstrate ROS induction upon IR of F1 cells. This effect occurs in F10, but to a lesser extent. This observation is consistent with radiosensitivity data for the two cell lines, where F1 is radiosensitive and F10 is radioresistant. (**b**–**e**) Elevated ROS does not necessarily correlate with AS. ROS data were plotted *versus* SI for each experimental treatment condition. Only some agents promote both senescence and ROS, whereas many others only elevated ROS, indicating that elevated ROS alone is not sufficient to induce senescence in either of these cell lines.

Thus, we screened SA-*β*-Gal at 660 nm (Figure 2a), AF at 525 nm (Figure 2b) and ROS at 450 nm emission (Figure 3) in cells treated with 0 or 5 Gy and redox-modulating compounds at doses documented to be effective in the literature (Supplementary Table 1). Median fluorescence intensities for duplicate microwell samples were calculated and averaged, and then SA-*β*-Gal and AF measurements were expressed as S:B ratios comparing the experimental sample *versus* a vehicle-only (DMSO) control for each group.

As observed in our initial studies with radiation alone, increases in SA-*β*-Gal were typically matched by a proportional increase in AF (Figures 2c–f). Linear regression analysis yielded correlation coefficients ranging from *R*^2^=0.75 (F1+0 Gy) to 0.94 (F10+5 Gy). In each plot, the single outlier is etoposide, which efficiently induced senescence in both cell lines, regardless of IR. Calculating SI for each condition in the screen yielded a wide range of scores for the hits modulating senescence, from 4.4 for F1 treated with AMA+0 Gy to 92.5 for F10 treated with etoposide+5 Gy (Figure 2g).

Examining senescence responses for the full range of compounds with and without IR, distinct patterns were observed for the two cell lines (Figures 2a, b and g). Based on SA-*β*-Gal and AF, treatment with 6 of 36 redox agents increased senescence in unirradiated B16-F1 cells, with etoposide >> trolox>TEMPOL>2-deoxyglucose>berberine>antimycin A. MnTBAP increased SA-*β*-Gal without appreciably increasing AF. Paradoxically, most of these compounds failed to increase senescence in irradiated F1 cells, except for etoposide and two compounds that displayed a minor synergistic effect with radiation, CDK4/6 inhibitor and mitomycin C. Unirradiated F10 cells were unresponsive to redox modulators other than etoposide. After IR of F10, three more compounds enhanced senescence, with etoposide >> berberine>rotenone>mitomycin C.

Following up on etoposide, we screened seven structurally diverse topoisomerase inhibitors that target topoisomerase II, topoisomerase I, or gyrase for senescence-inducing effects in unirradiated F10 cells. For each inhibitor, SA-*β*-Gal and AF increased proportionately (Supplementary Figure S3), leading to SI values that varied from close to background for the quinolone gyrase inhibitor moxifloxacin to a marked increase for the topoisomerase I inhibitor camptothecin. Notably, etoposide's effects fell in the middle of this group and below teniposide, suggesting it is representative of this class of drugs.

### LPO is correlated with the extent of AS induced by IR and topoisomerase inhibitors

To better understand potential links between redox-modulating compounds, topoisomerase inhibitors and senescence, we further examined the cellular ROS levels detected with the violet probe CV450 in the cytometric screen (Figure 3a). For the F1 cell line, while 5 Gy alone induced a measureable elevation in CV450 fluorescence, combining IR with 25 of the 36 redox-modulating compounds elevated ROS ≥2-fold more. Yet, most of these compounds failed to significantly increase SA-*β*-Gal or AF. For mitomycin C, although ROS levels detected after combined treatment were >25-fold higher than IR or mitomycin alone, this only yielded a twofold increase in SA-*β*-Gal or AF. Similarly, examining the correspondence between SI and ROS for each compound±5 Gy in each cell line demonstrated a weak correlation (Figures 3b–e, *R*^2^=0.37–0.65), with the one outlier being etoposide. These data raised the consideration that while ROS *per se* might not contribute to senescence, a specific form of oxidative damage might be a determinant.

**Figure 4:**
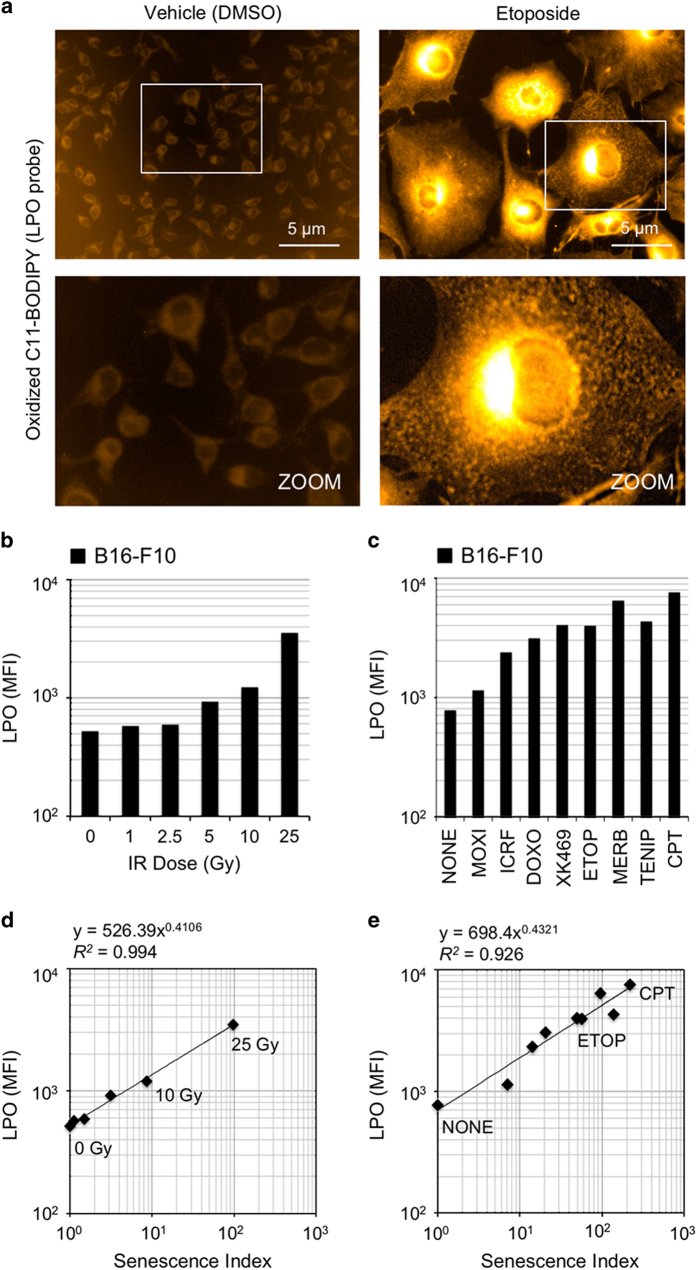
LPO is correlated with the extent of AS induced by IR and topoisomerase inhibitors. (**a**) Imaging of LPO in living cells using C11-BODIPY probe. B16-F10 cells were treated with either dimethyl sulfoxide (DMSO) vehicle (0.5%) or etoposide (2 *μ*M) for 96 h to induce senescence. Cells were imaged at ×20 magnification (upper row) using identical settings. An enlarged section of each image is shown (lower row) to demonstrate cellular distribution of the oxidized probe. Etoposide induces LPO and the oxidized lipid probe appears to accumulate in organelles, lipid droplets and the perinuclear region. (**b** and **c**) LPO induced by (**b**) IR and (**c**) topoisomerase inhibitors. Cells were treated, stained with C11-BODIPY and analyzed by flow cytometry. MFI for each sample condition is shown. Samples included 10 000 cells. (**d** and **e**) Strong correlation of LPO with SI for cells treated with (**d**) IR and (**e**) topoisomerase inhibitors. These results provide evidence that a combination of DNA damage and LPO may synergize to drive AS.

Based on subcellular location and chemical species, ROS can produce distinct patterns of modification of cellular macromolecules. We assessed damage to proteins by performing ELISA for advanced glycation end products (AGEs), immunostaining for oxidative DNA damage (8-OHdG) and analysis of LPO with BODIPY undecanoic acid (C11-BODIPY), a lipid probe that shifts emission from 590 to 510 nm upon oxidation. Although induction of AGEs and 8-OHdG varied among compounds that induced senescence (Supplementary Figures S4 and S5), LPO assays provided data of interest (Figure 4). F10 cells treated with etoposide exhibited marked LPO compared with vehicle (Figure 4a), as did F10 cells treated with IR doses from 0 to 25 Gy (Figure 4b), topoisomerase inhibitors (Figure 4c) and redox-modulating agents that induced senescence (Supplementary Figure S6). The extent of LPO induced by IR and topoisomerase inhibitors was strongly correlated to senescence (Figures 4d and e).

### LPO signaling and DNA damage synergize to induce AS

**Figure 5:**
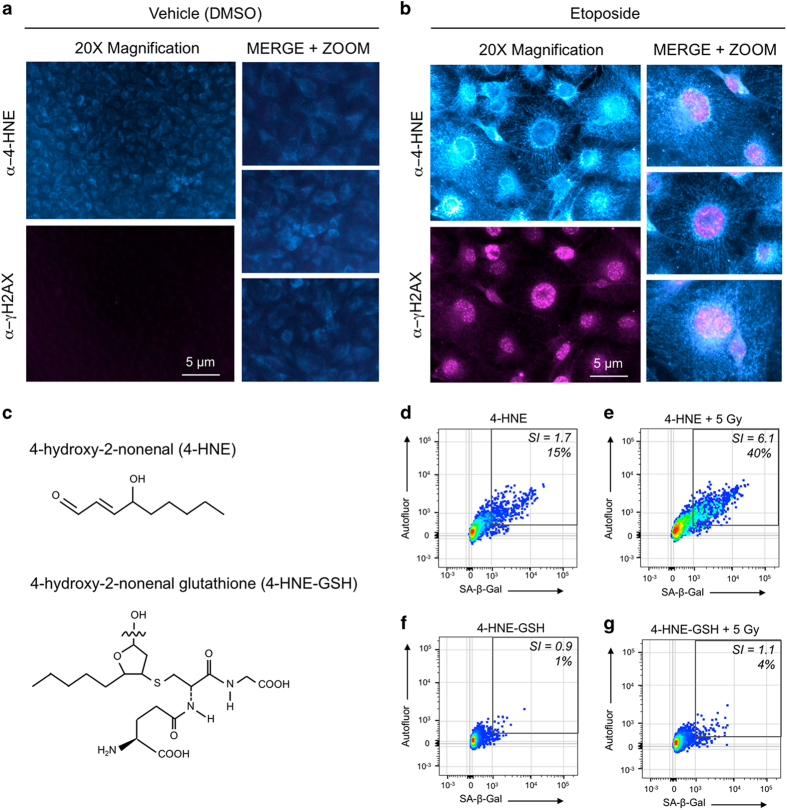
LPO signaling and DNA damage synergize to induce AS. (**a** and **b**) Double immunofluorescence staining for LPO marker 4-HNE and DNA damage marker *γ*H2AX (phospho-Ser139). (**a**) B16-F10 cells treated with DMSO only exhibited low levels of 4-HNE and negligible levels of *γ*H2AX at 96 h, whereas (**b**) cells treated with etoposide exhibited high levels of 4-HNE and *γ*H2AX foci at the same time point. Results indicate that DNA damage and LPO occur and persist in senescent cells induced by etoposide treatment. (**c**) Chemical structures of 4-HNE and 4-HNE-GSH. (**d**–**g**) 4-HNE and 4-HNE-GSH were applied to cells to observe senescence induction with and without IR. (**d**) 4-HNE induced senescence only in a minor fraction of cells (15%) and yielded a near-background SI of 1.7, whereas (**e**) IR potentiated the effects of 4-HNE, resulting in 40% senescent cells and an SI of 6.1. (**f** and **g**) 4-HNE-GSH did not induce senescence on its own or with IR, indicating that 4-HNE reactivity is directly responsible for the effects seen in (**d** and **e**).

To confirm that DNA damage and LPO were indeed both occurring and persisting within single senescent cells, we performed dual-immunofluorescence staining for nuclear foci of phosphorylated-H2AX (*γ*H2AX), a marker for DNA damage, and 4-hydroxy-2-nonenal (4-HNE) adducts, a marker for LPO, and the resulting accumulation of reactive aldehyde end products (Figures 5a and b). Cells were induced to senesce with etoposide or treated with vehicle only and incubated for 96 h. Cells were then fixed, probed with antibodies against the two targets and detected with fluorescent secondary antibodies. Control proliferating cells displayed no detectable DNA damage and a diffuse LPO signal (Figure 5a). In etoposide-treated senescent cells (Figure 5b), 4-HNE adducts were detected in the perinuclear and cytoplasmic regions and *γ*H2AX staining in the nuclei. Both 4-HNE and *γ*H2AX signals were present in >90% of etoposide-treated cells.

Reactive *α*,*β*-unsaturated aldehyde end products of LPO such as 4-HNE, acrolein and malondialdehyde readily form adducts with cellular thiols and other nucleophilic groups in proteins, nucleic acids and other targets, suggesting a potential role in the onset of senescence. Thus, we added chemically synthesized 4-HNE (Figure 5c) to F10 cells±IR (5 Gy) and examined the onset of senescence (Figures 5d and e). 4-HNE had little effect on its own, but efficiently promoted senescence when combined with 5 Gy (40%, SI=6.1). A non-reactive adduct, glutathione-trapped 4-HNE (4-HNE-GSH), failed to induce senescence on its own, or combined with IR (Figures 5f and g). These results suggest that LPO and DNA damage may synergize to promote AS and that 4-HNE is a major reactive aldehyde species contributing to the process.

**Figure 6:**
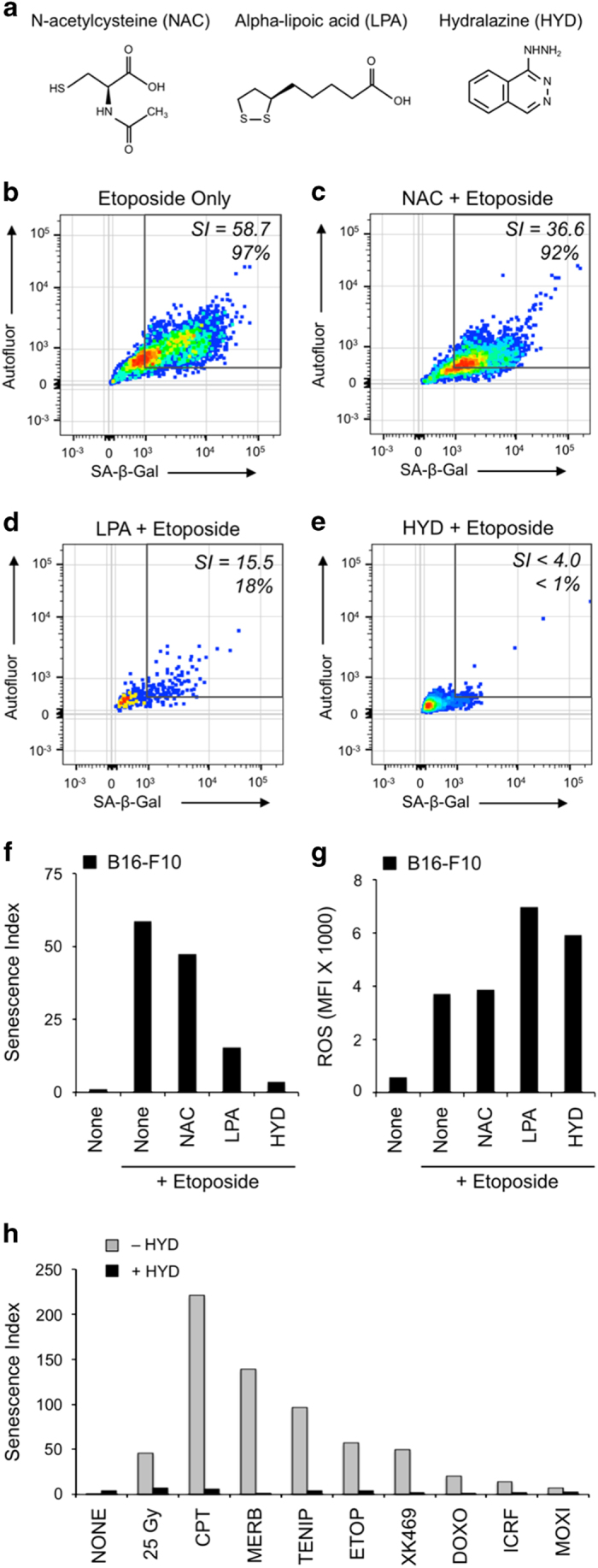
Lipid aldehyde scavenger HYD prevents the onset of AS. (**a**) Chemical structures of NAC, a general free-radical scavenging antioxidant, LPA, a general lipid antioxidant, and HYD HCl (HYD), an aldehyde scavenger. (**b**–**e**) Senescence assays were conducted for etoposide-treated cells that had been pretreated with compounds for 2 h, including (**b**) DMSO (0.5%), (**c**) NAC (1 mM), (**d**) LPA (5 mM) and (**e**) HYD (1 mM). Strikingly, HYD completely suppressed etoposide-induced senescence. (**f**) SI for each compound treatment shown in (**b**–**e**) indicating suppression of AS after etoposide by lipid antioxidants and aldehyde scavengers. (**g**) Median ROS signal for each compound treatment shown in (**b**–**e**). Notably, LPA and HYD increased ROS in etoposide-treated cells despite suppressing AS. (**h**) HYD blocked induction of senescence after IR or treatment with topoisomerase inhibitors.

To further examine whether reactive aldehyde end products of LPO such as 4-HNE may mediate the senescence-inducing effects of etoposide and other topoisomerase inhibitors, we tested the impact of lipid antioxidants and aldehyde scavengers (Figure 6a) on AS. The glutathione analog *N*-acetylcysteine (NAC) failed to block etoposide-induced senescence (92%, SI=36.6; Figures 6b and c). The lipid antioxidant *α*-lipoic acid (LPA) appeared to inhibit senescence (18%, SI=15.5; Figure 6d), and also reduced cell viability. However, HYD nearly completely blocked etoposide-induced senescence (<4%, SI<4.0, Figure 6e) without decreasing viability. Confirming that the senescence-suppressing effects of aldehyde-sequestering drugs were not simply due to an overall decrease in ROS and oxidative damage, we observed a reciprocal pattern between effects on SI and ROS (Figures 6f and g). Further implicating reactive aldehyde end products in AS, HYD was similarly effective in cells treated with otherwise senescence-inducing doses of various topoisomerase inhibitors or IR (Figure 6h and Supplementary Figure S7). In turn, HYD generally increased cell viability after topoisomerase treatment (Supplementary Figure S8a), but did so without substantially affecting total ROS (Supplementary Figure S8b).

## Discussion

In recent decades, prevailing models for cellular aging shifted away from ascribing a primary role to oxygen free radicals and metabolism, first to dependence on telomere erosion and then to unrepaired DNA damage anywhere on the chromosome. In turn, it is well accepted that it is DNA damage induced by chemotherapy or radiation that promotes TIS in cancer cells. Although a role for free radicals is still recognized in cell senescence, this is typically ascribed to induction of single- and double-strand DNA breaks. Here, in re-examining oxygen free radicals in TIS, our studies pointed us to a special role for LPO and aldehyde end products such as 4-HNE. While DNA is a major target for modification, 4-HNE readily forms adducts on proteins and other macromolecules, raising the possibility that signals independent of DNA damage may help drive tumor cells toward senescence.

Here, as a model for TIS, we have focused on the response of murine melanoma cells to the semisynthetic podophyllotoxin glucoside derivative etoposide (VP-16),^25^ a well-known topoisomerase II poison that is broadly used for cancer chemotherapy. Much like the anthracyclines,^26,27^ etoposide has long been studied not only as a topoisomerase II inhibitor^28^ but also as a source of free radicals with the potential to directly target cellular macromolecules.^29^ Among characterized reactions, P-450 metabolism of etoposide results in a reactive catechol that leads to a semiquinone radical, while tyrosinase can induce formation of a phenoxyl radical. Although etoposide is known to inhibit topoisomerase II activity by binding at the protein–DNA interface,^30^ there is also evidence that its mode of action may require metabolism to a radical and covalent protein modification.^31,32^ This raised the consideration whether the induction of senescence might reflect dual activities of this agent not only as a topoisomerase inhibitor but also as a source of free radicals.

Here, we screened redox modulators alone and in combination with low-dose radiation for their potential to induce AS in both B16-F1 and F10 cells. The F1 and F10 variant cell lines of the B16 melanoma tumor, syngeneic to the C57BL/6 mouse, were first selected in the 1970s^33^ and continue to be important tumor models not just for melanoma but also for metastasis, radiation response, immunotherapy and redox studies^34,35^ among others. The F1 variant is considered moderately ROS tolerant, radiosensitive and weakly metastatic, whereas F10 is known to be highly ROS tolerant, drug and radioresistant and highly metastatic,^36–40^ and as such the F10 tumor model represents a more difficult model to treat. Expression of the antioxidant enzyme manganese superoxide dismutase (SOD2, MnSOD) is significantly lower in F10 than in F1 cells.^41,42^ Potentially compensating for the MnSOD deficiency, levels of the antioxidant glutathione are higher in F10 cells.^43^ Our screen of redox modulators revealed distinct responses between the two cell lines. In the F1 cell line, multiple agents induced very high levels of ROS when combined with ionizing radiation, but the effect was less pronounced in the F10 cell line. Nonetheless, we observed only a weak correlation between ROS and induction and/or extent of senescence in each cell line.

Thus, we investigated specific types of oxidative damage in cells including protein glycation, DNA base oxidation and LPO. Correlative results between etoposide-induced senescence and LPO raised the hypothesis that DNA damage and LPO signaling might work together to promote senescence, and testing of LPO in senescent cells induced by IR and other topoisomerase inhibitors supported this hypothesis.

Much like the mechanism by which ROS promotes DNA damage, LPO is typically initiated by hydroxyl (HO^•^) radicals reacting with an unsaturated lipid to form a radical. Reaction with molecular oxygen can initiate propagation of free-radical chain reactions that terminate by producing reactive lipid aldehyde end products. Among these, 4-HNE can act as a second messenger to activate transcription factors p53, NF-*κ*B and Nrf2 and influence cell proliferation, cell cycle progression, survival, autophagy and/or senescence.^44^ En route to senescence, 4-HNE and other lipid aldehydes can modify and damage proteins,^45,46^ contributing to the formation of insoluble aggregates such as the autofluorescent product lipofuscin.^47,48^ LPO end products can also modify DNA and affect gene expression and genome stability.^49,50^ Further supporting our hypothesis regarding synergy between DNA damage and LPO signaling, we observed that exposure to the LPO signaling aldehyde 4-HNE had little senescence effect on its own, yet this agent efficiently induced senescence in F10 cells when combined with IR. Using HYD to scavenge lipid aldehyde species prevented induction of senescence by IR, etoposide, or other topoisomerase inhibitors including camptothecin, merbarone, teniposide, XK469 and doxorubicin.

Taken together, the data generated by this study support the hypothesis that it is not purely ROS, DNA damage, or a combination of the two that is sufficient to induce cellular senescence. More specifically, LPO and its reactive aldehyde end products such as 4-HNE likely have a key role in induction of AS, particularly by topoisomerase inhibitors.

## Materials and Methods

### Materials

B16-F1 and B16-F10 cells were obtained from American Type Culture Collection (ATCC, Manassas, VA, USA). Topoisomerase inhibitors were purchased from Sigma-Aldrich (St. Louis, MO, USA; camptothecin, merbarone, teniposide, etoposide, XK469, ICRF-193) and Cayman Chemical (Ann Arbor, MI, USA; doxorubicin, moxifloxacin). NAC, LPA and HYD HCl were from Sigma-Aldrich. Fluorescent probes were from Life Technologies (Waltham, MA, USA; DDAOG, C11-BODIPY, CellROX Green, CellTrace Violet), eBioscience (San Diego, CA, USA; CV450) and Sigma-Aldrich (DAPI). Bafilomycin A1 was from Research Products International (Mount Prospect, IL, USA). Primary antibodies were from Millipore (Billerica, MA, USA; anti-*γ*H2AX, clone JBW301) or Abcam (Cambridge, UK; anti-4-HNE, anti-OHdG). Fluorescent secondary antibodies were obtained from Thermo Pierce (Waltham, MA, USA). Cell culture reagents were from Life Technologies (DMEM, pen-strep solution, trypsin-EDTA) and Gemini Biosciences (West Sacramento, CA, USA; fetal bovine serum, stabilized l-glutamine).

### Cell culture, compound treatments and γ-IR

Cells were maintained in culture medium consisting of DMEM supplemented with 10% fetal bovine serum, 4 mM stabilized l-glutamine and 100 U/100 *μ*g per ml penicillin/streptomycin. Low passage (*P*<10) cells were used. Cells were seeded at a density of 5×10^3^ cells per cm^2^ in culture flasks or plates and incubated overnight before treatment. Compound agents were diluted in DMSO or H_2_O. The final solvent concentration did not exceed 0.5%. Topoisomerase inhibitors were applied to cells for 96 h. The panel of 36 redox modulators as well as NAC, lipoic acid and HYD were applied in culture media for 2 h before induction of senescence by IR or compound treatment and then left on cells for the duration of the experiment. γ-IR was carried out using a ^137^Cs γ-irradiator (8.1 Gy/min); cells receiving 0 Gy were mock irradiated.

### Flow cytometric senescence assay

For SA-*β*-Gal screening analysis in 96-well plates, media were exchanged to 100 *μ*l culture medium containing 1 *μ*M bafilomycin A1. After 30 min at 37 °C (without CO_2_), 10 *μ*g/ml DDAOG was added and cells were incubated for 1 h at 37 °C. Wells were washed once with DPBS (Ca^2+^ and Mg^2+^ free) and then treated with 100 *μ*l prewarmed trypsin-EDTA (0.25%) for 5 min at 37 °C. Detachment was confirmed by phase-contrast microscopy, and 100 *μ*l of culture medium was added to neutralize the trypsin. One micromolar of CV450 was added for 30 min at 37 °C, and microplates were analyzed by flow cytometry (BD Fortessa HTS with FACSDiva software, Becton Dickinson, Franklin Lakes, NJ, USA). Calibration particles (Ultra Rainbow; SpheroTech, Lake Forest, IL, USA) were used to standardize cytometer settings across runs. A maximum threshold of 10 000 events or 100 *μ*l volume was analyzed per microwell at a flow rate of 2 *μ*l/s. Data were acquired using the following excitation lasers and detection optics: CV450/ROS, 405 nm laser, 450/50 nm detector; cellular AF, 488 nm laser, 525/50 nm detector; DDAOG/SA-*β*-Gal, 640 nm laser, 670/30 detector. Raw data files (.fcs) were exported and analyzed using flow cytometric analysis software (FlowJo; TreeStar, Ashland, OR, USA). For SA-*β*-Gal analysis in six-well plates, the stated workflow was followed with the following modifications: (1) trypsinization was carried out as a first step to release cells from plates, (2) cells were stained in microtubes, (3) 1 ml staining volume was used and (4) a minimum threshold of 10 000 events were acquired for all samples.

### Oxidative damage assays

To assay ROS, cells were detached with trypsin-EDTA, washed and incubated with 1 *μ*M CV450 or 10 *μ*M CellROX Green in the culture medium for 30 min at 37 °C. Cells were washed and analyzed by flow cytometry using appropriate optical settings (CV450, 405 nm laser, 450/50 nm detector; CellROX Green, 488 nm laser, 525/50 nm detector). To assay LPO, C11-BODIPY was added at 10 *μ*M in complete media for 30 min at 37 °C. Cells were then washed with DPBS and imaged using wide-field epifluorescent microscopy using an appropriate filter set (bandpass 525/50 nm) to image the oxidized form of the probe. For flow cytometry, cells were treated with C11-BODIPY as described, detached with trypsin-EDTA, washed and analyzed by flow cytometry using a 488 nm excitation laser and 525/15 nm detector to collect data on the oxidized form of the C11-BODIPY probe. Immunofluorescence staining of oxidized DNA (8-OHdG) was performed by fixing cells grown on sterile glass coverslips in 4% paraformaldehyde for 10 min, followed by permeabilization with 0.05% Triton-X-100 in 1× DPBS (PBS-TX) for 10 min, and blocking with 5% BSA in DPBS for 30 min at 24 °C. Then, 1 *μ*g/ml 8-OHdG antibody in 1% BSA-DPBS was added for 18 h at 4 °C, followed by washing 3×5 min with PBS-TX. Later, 10 *μ*g/ml anti-goat-Alexa Fluor 488 conjugate (Thermo Pierce) was added for 1 h at 4 °C, followed by washing 3×5 min with PBS-TX, and counterstaining of cell nuclei with 1 *μ*g/ml DAPI for 10 min at room temperature. Coverslips were rinsed with DPBS, affixed to glass slides using mounting medium (ProLong Gold AntiFade; Life Technologies) and slides were dried overnight. Slides were imaged at 40× using a Zeiss Axiovert 40 microscope (Carl Zeiss, Oberkochen, Germany) using a 525/50 nm bandpass filter set. Competitive ELISA assays for AGEs were performed with the AGE competitive ELISA Assay Kit (Cell Biolabs, San Diego, CA, USA) according to the manufacturer's directions. Briefly, 2 *μ*g of cell lysates prepared as stated above were added to microwells precoated with AGE conjugate. Anti-AGE primary antibody was added, followed by three washes, and then secondary antibody-HRP conjugate and three washes. Wells were developed with colorimetric HRP substrate TMB (3,3′,5,5′-tetramethylbenzidine), stopped and absorbance at 450 nm was determined using a microplate reader (Tecan Safire, Mannedorf, Switzerland). AGE levels were determined by comparison with a standard curve of AGE-BSA.

### Immunofluorescence imaging of 4-HNE and *γ*H2AX

Dual-immunofluorescence staining of 4-HNE and *γ*H2AX was performed by fixing cells grown on sterile glass coverslips in 4% paraformaldehyde for 10 min, followed by permeabilization with 0.05% Triton X-100 in 1× DPBS (PBS-TX) for 10 min, and blocking with 5% BSA in DPBS for 30 min at 24 °C. Then, anti-4-HNE (goat, 1/1000× dilution) and anti-*γ*H2AX (mouse, 1 *μ*g/ml) were added in 1% BSA-DPBS for 18 h at 4 °C, followed by washing 3×5 min with PBS-TX. Anti-goat-DyLight-594 and anti-mouse-DyLight 650 conjugate (1/5000× dilution each) were added for 30 min at 24 °C in 5% BSA-DPBS, followed by washing 3×5 min with PBS-TX, and counterstaining of cell nuclei with 1 *μ*g/ml DAPI for 10 min at room temperature. Coverslips were rinsed with DPBS, affixed to glass slides using mounting medium (ProLong Gold AntiFade; Life Technologies) and slides were dried overnight. Slides were imaged at 40× using a Zeiss Axiovert 40 microscope (Carl Zeiss) equipped with a monochromatic camera using a 590 nm long-pass filter set to image the anti-4-HNE labeled with anti-goat-DyLight-594 and a 630-nm-long-pass filter set to image the anti-*γ*H2AX labeled with anti-mouse-DyLight 650. Image settings were set using untreated cells and were unchanged throughout image acquisition. Images were pseudocolored using FIJI software (NIH, Bethesda, MD, USA).

### Brightfield imaging of SA-*β*-Gal using X-Gal staining

Cells grown in culture dishes were fixed for 4 min in a solution of 4% paraformaldehyde+0.5% glutaraldehyde freshly prepared in DPBS and then washed two times with DPBS. The X-Gal staining solution, consisting of 1 mg/ml X-Gal in a pH 6.0 phosphate buffer with 5 mM ferricyanide, 5 mM ferrocyanide and 2 mM MgCl_2_, was added and the cells were incubated for 18 h at 37 °C without CO_2_. Cells were washed once with PBS and imaged using PlasDIC brightfield settings and ×20 magnification on a Zeiss Axiovert 40 microscope (Carl Zeiss) with a color camera. White balance was set using background of each image. Images were analyzed using FIJI software.

### Cell proliferation assays

For cell proliferation assays, 5 *μ*M CellTrace Violet in DPBS was added to adherent cells for 15 min at 37 °C, and then cells were washed with DPBS and returned to media. Cells were incubated for 24 to 96 h, detached with trypsin, washed and analyzed by flow cytometry using 405 nm laser and 450/50 nm detector. Ten thousand events were collected per sample, and the raw data were exported (.fcs) and analyzed using the FlowJo software.

## Abbreviations

4-HNE: 4-hydroxy-2-nonenal
4-HNE-GSH: 4-hydroxy-2-nonenal glutathione
AF: autofluorescence
AS: accelerated senescence
C_12_-FDG: 5-dodecanoyl-aminofluorescein-di-*β*-d-galactopyranoside
CV450: Calcein Violet 450 AM
DDAO: 7-hydroxy-9*H*-(1,3-dichloro-9,9-dimethylacridin-2-one)
DDAOG: 9*H*-(1,3-dichloro-9,9-dimethylacridin-2-one-7-yl)-*β*-d-galactopyranoside
HYD: hydralazine HCl
LPO: lipid peroxidation
MFI: median fluorescence intensity
ROS: reactive oxygen species
RS: replicative senescence
S:B: signal to background ratio
SA-*β*-Gal: senescence-associated-*β*-galactosidase
SI: senescence index
TIS: therapy-induced senescence
X-Gal: 5-bromo-4-chloro-3-indolyl-*β*-d-galactopyranoside.
