# Supplementary material for: Modulation of therapy-induced senescence by reactive lipid aldehydes
Source: Cell Death Discov. 2016 Jul 4;2:16045–. doi: 10.1038/cddiscovery.2016.45 (PMC4957821; doi:10.1038/cddiscovery.2016.45)
Supplement: Supplementary Information [file cddiscovery201645-s1.doc]

**Supplementary Information**

|  | **Compound** | **Conc** | **Source** | **CAS #** | **Ref** |
| --- | --- | --- | --- | --- | --- |
| **1** | 2-deoxy-D-glucose (2-DG) | 4 mM | Sigma-Aldrich | 154-17-6 | S1 |
| **2** | 3-bromopyruvate (3-BP) | 10 µM | Sigma-Aldrich | 1113-59-3 | S2 |
| **3** | Alloxan monohydrate | 1 mM | Sigma-Aldrich | 2244-11-3 | S3 |
| **4** | Antimycin A (AMA) | 0.2 µM | Sigma-Aldrich | 1397-4-0 | S4 |
| **5** | Ascorbate, L-, sodium salt | 200 µM | Acros Organics | 134-03-2 | S5 |
| **6** | Berberine, hemisulfate salt | 30 µM | Sigma-Aldrich | 633-66-9 | S6 |
| **7** | Butlyated hydroxyanisole (BHA) | 100 µM | Sigma-Aldrich | 25013-16-5 | S7 |
| **8** | Butyrate, sodium salt | 4 mM | Sigma-Aldrich | 156-54-7 | S8 |
| **9** | Caffeine | 1 mM | Sigma-Aldrich | 58-08-2 | S9 |
| **10** | Capsaicin | 100 µM | Santa Cruz Biotech | 404-86-4 | S10 |
| **11** | CDK4/6 inhibitor (PD-0332991) | 1 µM | Selleck Chemical | 827022-32-2 | S11 |
| **12** | Curcumin | 10 µM | CalBioChem | 458-37-7 | S12 |
| **13** | Epigallocatachin gallate (EGCG) | 1 µM | Sigma-Aldrich | 989-51-5 | S13 |
| **14** | Etoposide | 1.7 µM | Sigma-Aldrich | 33419-42-0 | S14 |
| **15** | EUK 134 | 20 µM | Cayman Chemical | 81065-76-1 | S15 |
| **16** | Genistein | 10 µM | Sigma-Aldrich | 446-72-0 | S16 |
| **17** | Glutathione, L-, reduced (GSH) | 3.25 µM | Sigma-Aldrich | 70-18-8 | S17 |
| **18** | Hydrogen peroxide (H2O2) | 1 µM | FisherBrand Chemical | 7722-84-1 | S18 |
| **19** | Menadione | 12.5 µM | Sigma-Aldrich | 58-27-5 | S19 |
| **20** | Metformin hydrochloride | 1 mM | Sigma-Aldrich | 1115-70-4 | S20 |
| **21** | Mitomycin C | 0.2 µM | Sigma-Aldrich | 50-07-7 | S21 |
| **22** | MitoTEMPO | 0.1 µM | Sigma-Aldrich | 1334850-99-5 | S22 |
| **23** | MnTBAP chloride | 100 µM | Enzo Diagnostics | 55266-18-7 | S23 |
| **24** | N-acetylcysteine (NAC) | 1 mM | Sigma-Aldrich | 616-91-1 | S24 |
| **25** | Nrf2 inhibitor (IM3829) | 5 µM | Matrix Scientific | 76256-34-4 | S25 |
| **26** | Orcinol (5-methylresorcinol) | 140 µM | Sigma-Aldrich | 504-15-4 | S26 |
| **27** | Paraquat dichloride hydrate | 50 µM | Sigma-Aldrich | 75365-73-0 | S27 |
| **28** | Phloretin | 50 µM | Sigma-Aldrich | 60-82-2 | S28 |
| **29** | Quercetin 3-O-galactoside | 10 µM | Santa Cruz Biotech | 482-36-0 | S29 |
| **30** | Rapamycin | 0.1 µM | Sigma-Aldrich | 53123-88-9 | S30 |
| **31** | Resveratrol | 10 µM | Sigma-Aldrich | 501-36-0 | S31 |
| **32** | Rho Kinase Inhibitor III (Rockout) | 25 µM | Santa Cruz Biotech | 7272-84-6 | S32 |
| **33** | Rotenone | 0.6 µM | Sigma-Aldrich | 83-79-4 | S33 |
| **34** | Rutin | 200 µM | Sigma-Aldrich | 250249-75-3 | S34 |
| **35** | TEMPOL | 5 mM | Sigma-Aldrich | 2226-96-2 | S35 |
| **36** | Trolox | 1 mM | Acros Organics | 53188-07-1 | S36 |

**Supplementary Table 1. Compounds used in the study.**

Redox modulating compounds, concentrations used, source, CAS number and references are noted in the table. For references, please see **Supplementary References**.

**
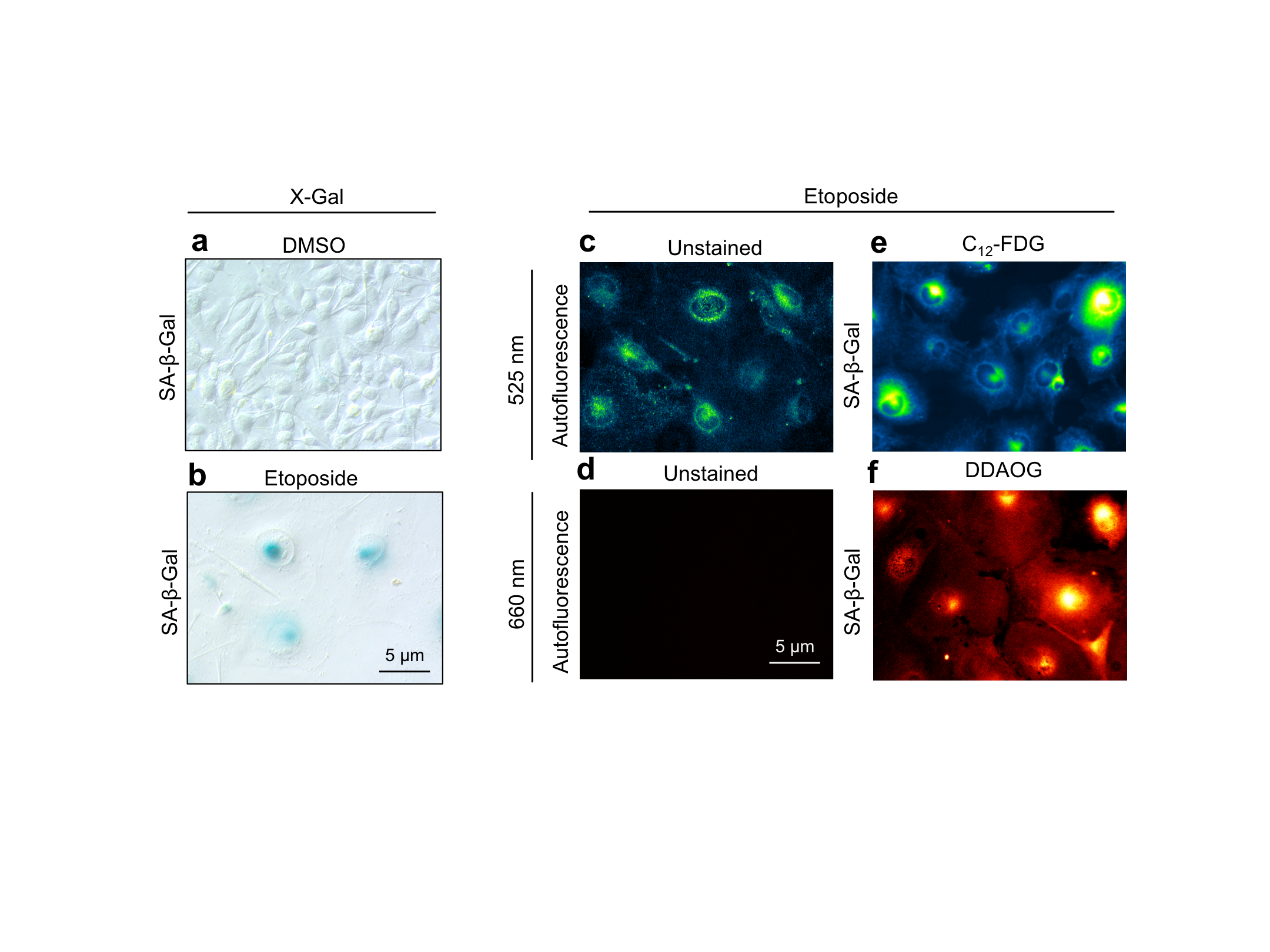
**

**Supplementary Figure S1.** **DDAOG is a sensitive and specific stain for SA-β-Gal.**

**(a, b)** Conventional staining for SA-β-Gal using X-Gal showing etoposide-induced senescence in B16-F10 cells. Senescent cells exhibit blue perinuclear staining.

**(c, d)** Evaluation of background fluorescence of unstained, etoposide-treated live cells in either the green (525 nm) or near-infrared (660 nm) emission channel. Background signal from lipofuscin is high in the green emission channel and negligible in the near-infrared channel (exposure time 2 s for each image).

**(d, e)** Fluorescent staining for SA-β-Gal in etoposide-treated cells using either C12-FDG (515 nm) or DDAOG (660 nm). Staining distribution is similar for both probes, indicating that DDAGO detects SA-β-Gal in a similar manner to C12-FDG. However, because background signal is high in the green emission channel used to detect C12-FDG, results may be confounded using this probe.


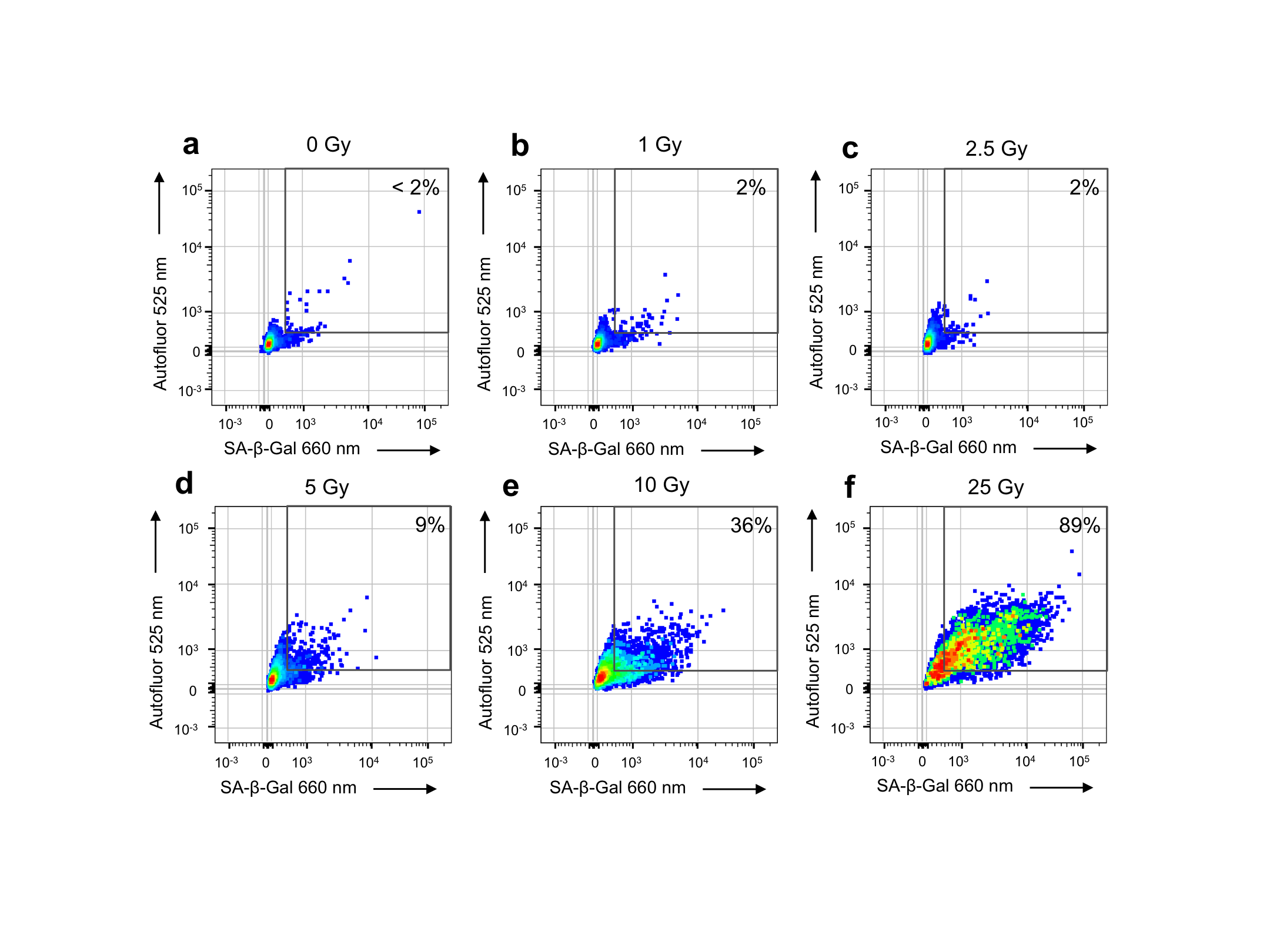


**Supplementary Figure S2. IR-induced senescence dose curve for B16-F1 cell line.**

Flow cytometric dot plots indicating increasing senescence in B16-F1 cells with increasing IR dose, measuring SA-β-Gal and autofluorescence in each cell.


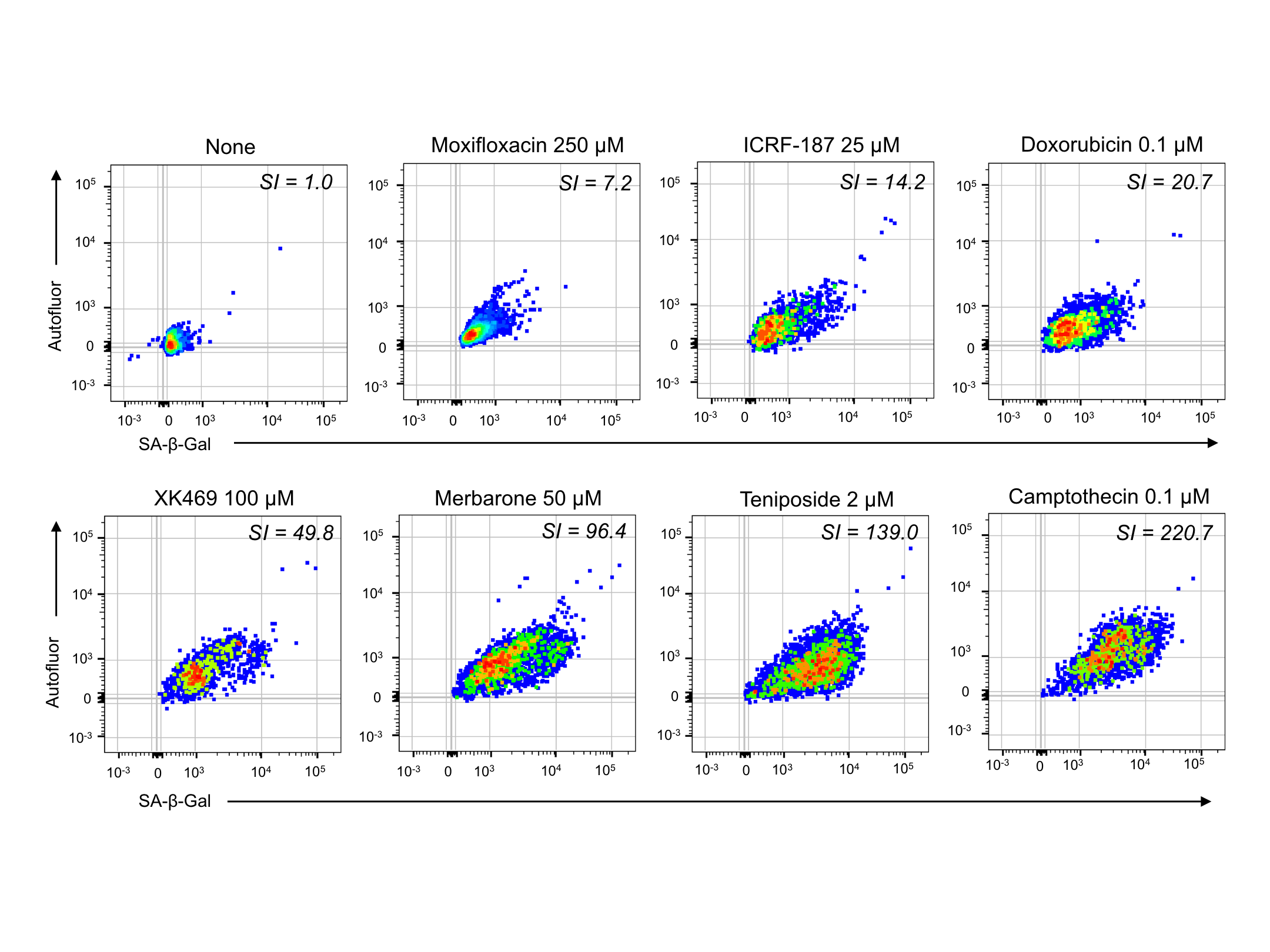


**Supplementary Figure S3. Accelerated senescence induction by diverse topoisomerase inhibitors.**

Flow cytometric senescence assay of B16-F10 cells treated with topoisomerase inhibitors at effective doses drawn from literature and confirmed on this cell line. Etoposide (**Fig. 5i**) displayed an intermediate effect (SI = 58.7) compared to these agents.

**
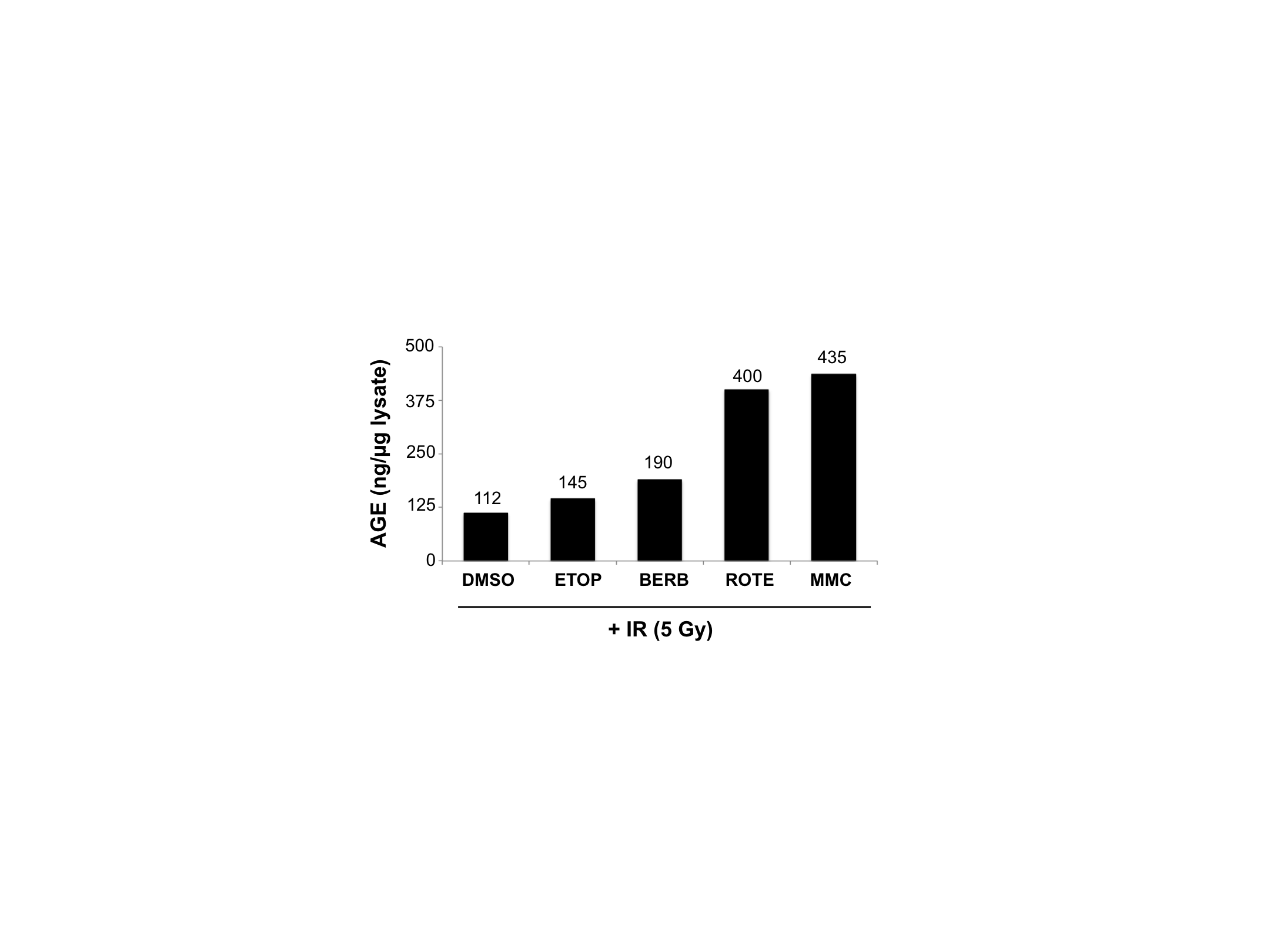
**

**Supplementary Figure S4. Advanced Glycation End-products (AGE) ELISA after treatment with senescence-inducing agents and radiation.**

Detection of advanced glycation end products (AGEs) by competitive ELISA assay was performed on lysates of B16-F10 cells treated with 5 Gy and DMSO or agents that induced senescence alone or in combination with radiation. AGEs failed to correlate with senescence induction.

**
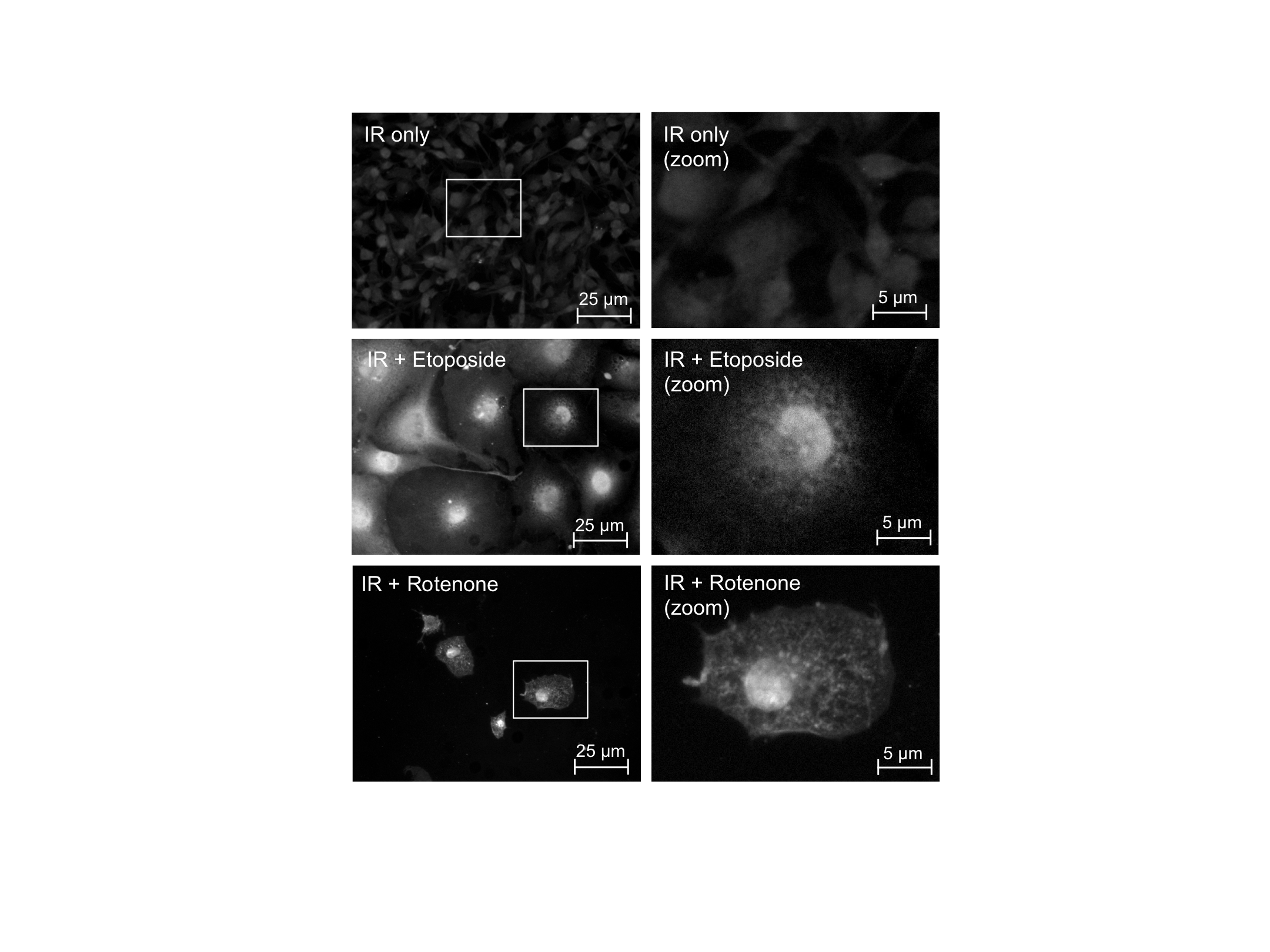
**

**Supplementary Figure S5. Oxidized DNA (8-OHdG) immunostaining.**

Immunofluorescence detection of oxidative DNA damage with anti-8-OHdG (8-hydroxy-2’-deoxyguanosine) revealed a lack of nuclear or cytoplasmic staining in 5 Gy irradiated B16-F10 cells but distinct staining after radiation and etoposide or rotenone. Nuclear staining suggests oxidative damage to chromosomal DNA while cytoplasmic staining may indicate oxidative damage to mitochondrial DNA. Anti-8-OHdG staining was similar to 5 Gy background after 5 Gy plus berberine or mitomycin C (data not shown), despite induction of senescence.

**
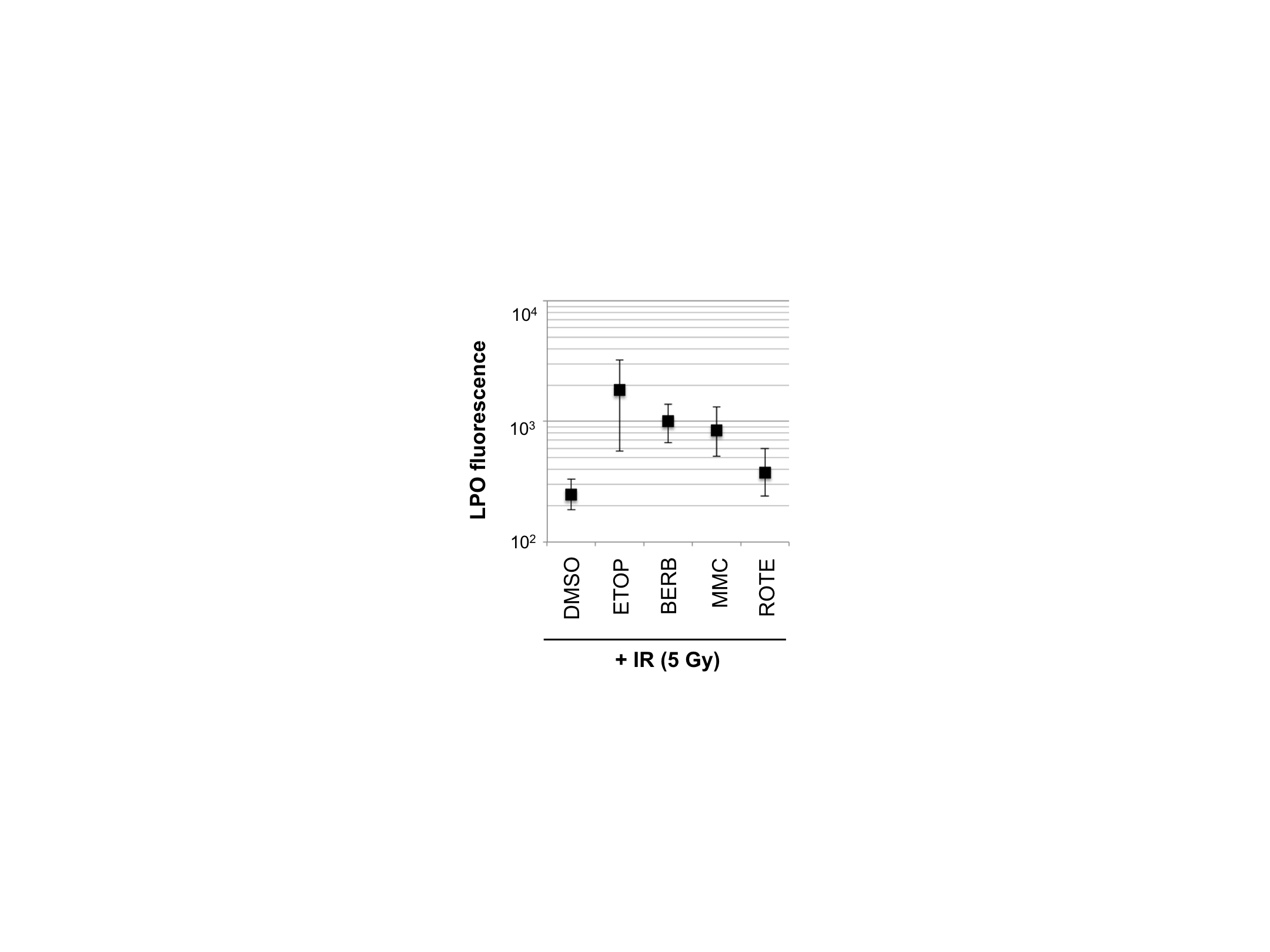
**

**Supplementary Figure S6. Lipid peroxidation is induced by senescence screening hits.**

Lipid peroxidation (LPO) was assayed in cells treated with 5 Gy and DMSO or agents observed to induce senescence in irradiated B16-F10 cells. Median fluorescence intensity of sample populations comprised of ≥ 5 000 viable cells are indicated by symbols. Error bars indicate 25th and 75th percentile of cellular staining distributions. LPO levels correlated with relative induction of senescence.


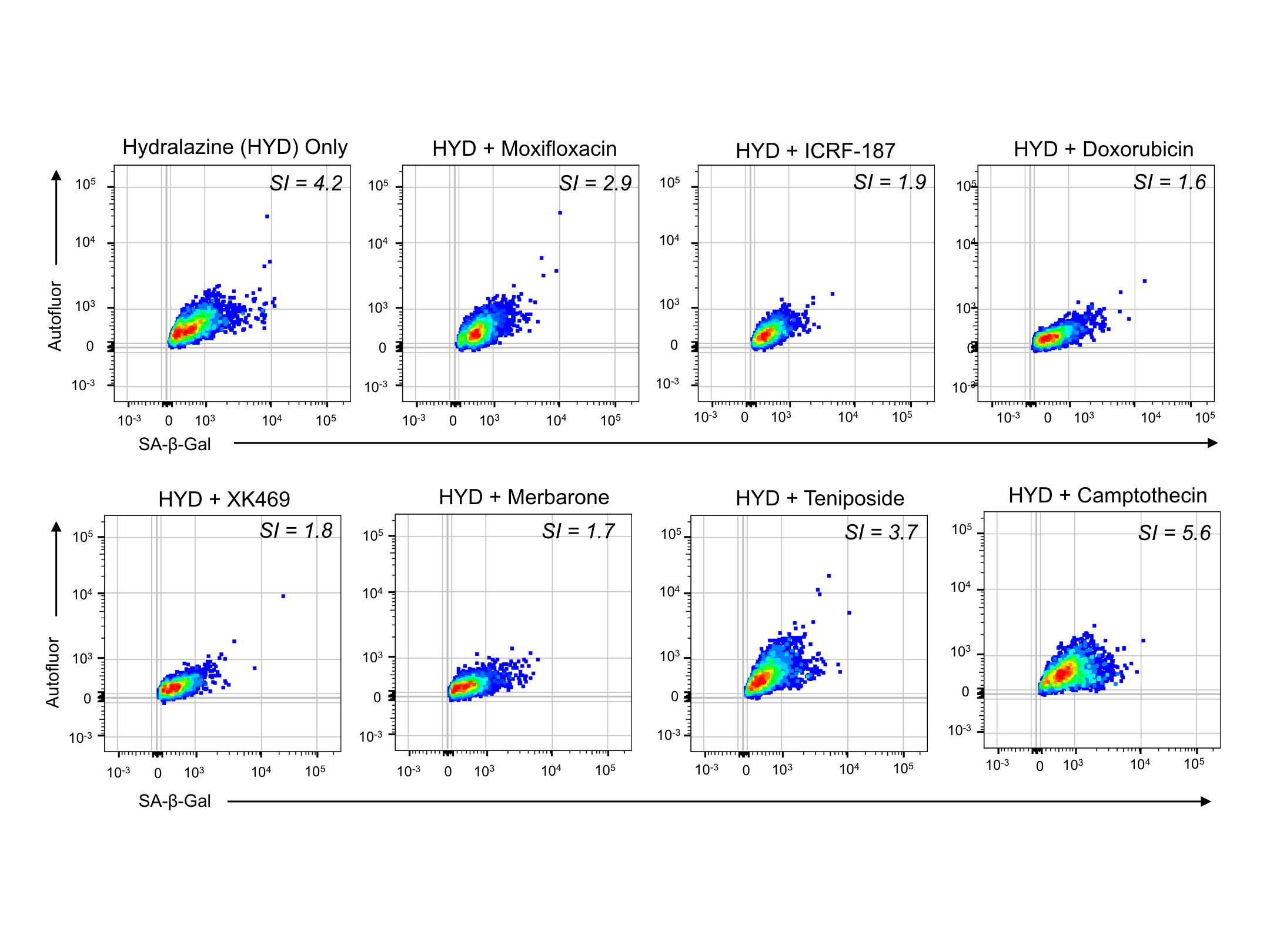


**Supplementary Figure S7. Aldehyde sequestering drug hydralazine blocks senescence induction by topoisomerase inhibitors.**

Flow cytometric senescence assay of B16-F10 cells incubated for 2 hours with 1 mM hydralazine before treatment with topoisomerase inhibitors. Hydralazine suppressed both autofluorescence and SA-β-Gal induced by each topoisomerase inhibitor, as compared to **Supplementary Fig.** **S6**.


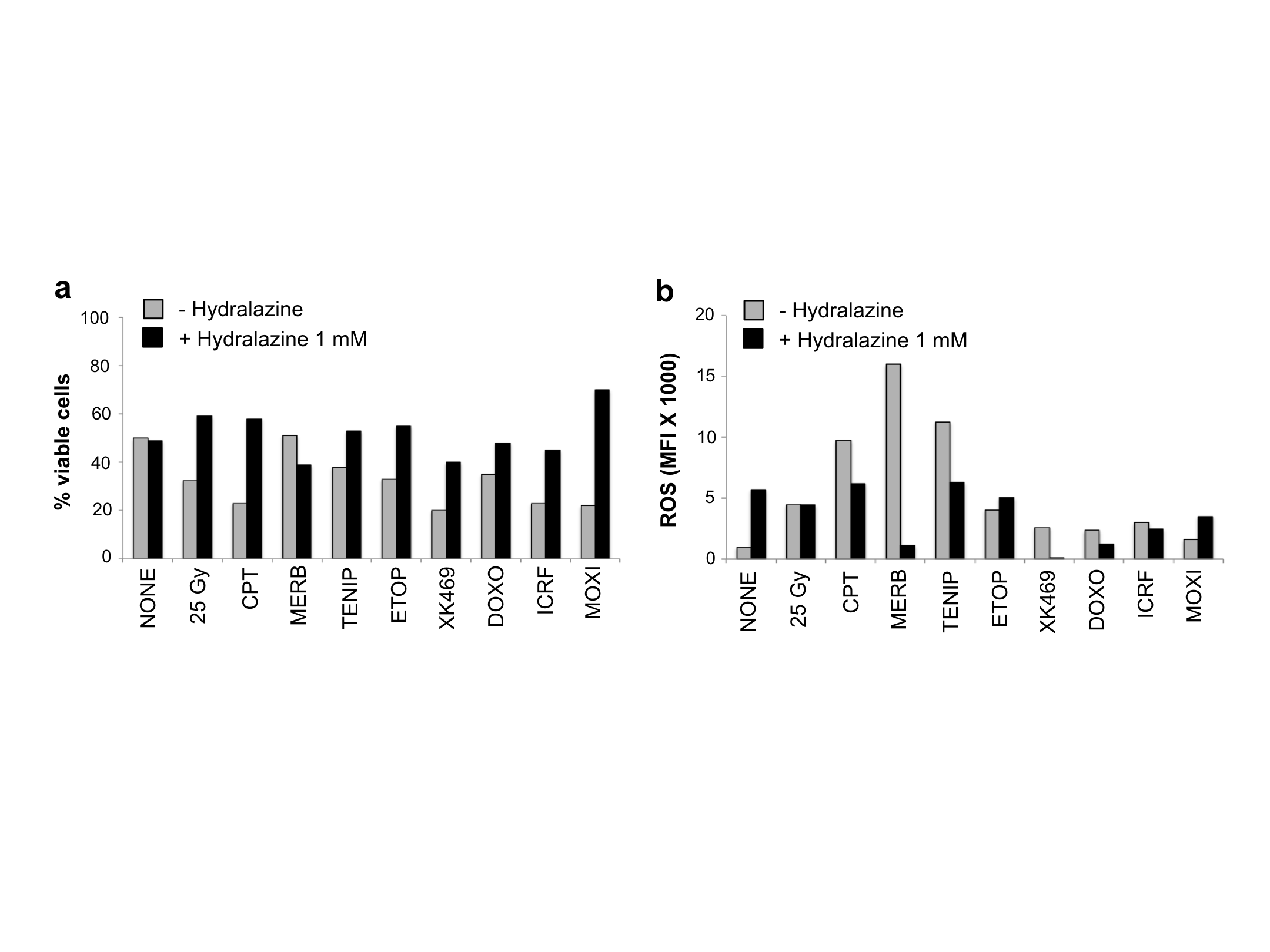


**Supplementary Figure S8. Hydralazine treatment protects cell viability and has variable effects on cellular ROS.**

**(a)** Cell viability was measured by flow cytometry for cells treated with radiation or topoisomerase inhibitors +/- hydralazine. Hydralazine was non-toxic on its own and conferred moderate protection against cell death in the majority of treatments tested.

**(b)** ROS was measured in viable cells by flow cytometry for cells treated with radiation or topoisomerase inhibitors +/- hydralazine. Median ROS fluorescence is shown. Results demonstrate variable effects on ROS. On its own, hydralazine elevated ROS. With IR 25 Gy, hydralazine had no observable effect *vs*. IR alone. With topoisomerase inhibitors, hydralazine lowered cellular ROS in some cases (camptothecin, merbarone, teniposide, XK469), but slightly elevated ROS in others (etoposide, moxifloxacin). These results are consistent with a minor role for ROS in senescence.

**Supplementary References**

1. Liou J-Y. 2-Deoxyglucose Enhances the Chemosensitivity of B16-F10 Melanoma Cells to Cisplatin (Thesis) 2013.

2. Qin J-Z, Xin H, Nickoloff B. 3-Bromopyruvate induces necrotic cell death in sensitive melanoma cell lines. Biochemical and Biophysical Research Communications. 2010;396(2):495-500.

3. Diab RAH, Fares M, Abedi-Valugerdi M, Kumagai-Braesch M, Holgersson J, Hassan M. Immunotoxicological effects of streptozotocin and alloxan: *In vitro* and *in vivo* studies. Immunology Letters. 2015;163(2):193-8.

4. Park WH, Han YW, Kim SH, Kim SZ. An ROS generator, antimycin A, inhibits the growth of HeLa cells via apoptosis. Journal of Cellular Biochemistry. 2007;102(1):98-109.

5. Kang JS, Cho D, Kim YI, Hahm E, Kim YS, Jin SN, et al. Sodium ascorbate (vitamin C) induces apoptosis in melanoma cells via the down‐regulation of transferrin receptor dependent iron uptake. Journal of Cellular Physiology. 2005;204(1):192-7.

6. Hur JM, Hyun MS, Lim SY, Lee WY, Kim D. The combination of berberine and irradiation enhances anti‐cancer effects via activation of p38 MAPK pathway and ROS generation in human hepatoma cells. Journal of Cellular Biochemistry. 2009;107(5):955-64.

7. Saito M, Sakagami H, Fujisawa S. Cytotoxicity and apoptosis induction by butylated hydroxyanisole (BHA) and butylated hydroxytoluene (BHT). Anticancer Research. 2003;23(6c):4693-701.

8. Romanov VS, Abramova MV, Svetlikova SB, Bykova TV, Zubova SG, Aksenov ND, et al. p21Waf1 is required for cellular senescence but not for cell cycle arrest induced by the HDAC inhibitor sodium butyrate. Cell Cycle. 2010;9(19):3975-85.

9. Noppe G, Dekker P, de Koning-Treurniet C, Blom J, van Heemst D, Dirks RW, et al. Rapid flow cytometric method for measuring senescence associated β-galactosidase activity in human fibroblasts. Cytometry Part A. 2009;75A(11):910-6.

10. Silverberg JI, Patel M, Brody N, Jagdeo J. Caffeine protects human skin fibroblasts from acute reactive oxygen species-induced necrosis. Journal of Drugs and Dermatology. 2012;11(11):1342-6.

11. Jun H-S, Park T, Lee CK, Kang MK, Park MS, Kang HI, et al. Capsaicin induced apoptosis of B16-F10 melanoma cells through down-regulation of Bcl-2. Food and Chemical Toxicology. 2007;45(5):708-15.

12. Franco J, Witkiewicz AK, Knudsen ES. CDK4/6 inhibitors have potent activity in combination with pathway selective therapeutic agents in models of pancreatic cancer. Oncotarget. 2014;5(15):6512.

13. Mosieniak G, Adamowicz M, Alster O, Jaskowiak H, Szczepankiewicz AA, Wilczynski GM, et al. Curcumin induces permanent growth arrest of human colon cancer cells: link between senescence and autophagy. Mechanisms of Ageing and Development. 2012;133(6):444-55.

14. Ellis LZ, Liu W, Luo Y, Okamoto M, Qu D, Dunn JH, et al. Green tea polyphenol epigallocatechin-3-gallate suppresses melanoma growth by inhibiting inflammasome and IL-1β secretion. Biochemical and Biophysical Research Communications. 2011;414(3):551-6.

15. Robles SJ, Buehler PW, Negrusz A, Adami GR. Permanent cell cycle arrest in asynchronously proliferating normal human fibroblasts treated with doxorubicin or etoposide but not camptothecin. Biochemical Pharmacology. 1999;58(4):675-85.

16. Limoli CL, Giedzinski E, Baure J, Doctrow SR, Rola R, Fike JR. Using superoxide dismutase/catalase mimetics to manipulate the redox environment of neural precursor cells. Radiation Protection Dosimetry. 2006;122(1-4):228-36.

17. Record IR, Broadbent JL, King RA, Dreosti IE, Head RJ, Tonkin AL. Genistein inhibits growth of B16 melanoma cells in vivo and in vitro and promotes differentiation in vitro. International Journal of Cancer. 1997;72(5):860-4.

18. Franzini M, Corti A, Lorenzini E, Paolicchi A, Pompella A, De Cesare M, et al. Modulation of cell growth and cisplatin sensitivity by membrane γ-glutamyltransferase in melanoma cells. European Journal of Cancer. 2006;42(15):2623-30.

19. Chen QM, Tu VC, Liu J. Measurements of hydrogen peroxide induced premature senescence: senescence-associated β-galactosidase and DNA synthesis index in human diploid fibroblasts with down-regulated p53 or Rb. Biogerontology. 2000;1(4):335-9.

20. Liu Z, Tao X, Zhang C, Lu Y, Wei D. Protective effects of hyperoside (quercetin-3-o-galactoside) to PC12 cells against cytotoxicity induced by hydrogen peroxide and tert-butyl hydroperoxide. Biomedicine & Pharmacotherapy. 2005;59(9):481-90.

21. Loor G, Kondapalli J, Schriewer JM, Chandel NS, Vanden Hoek TL, Schumacker PT. Menadione triggers cell death through ROS-dependent mechanisms involving PARP activation without requiring apoptosis. Free Radical Biology and Medicine. 2010;49(12):1925-36.

22. Janjetovic K, Harhaji-Trajkovic L, Misirkic-Marjanovic M, Vucicevic L, Stevanovic D, Zogovic N, et al. In vitro and in vivo anti-melanoma action of metformin. European Journal of Pharmacology. 2011;668(3):373-82.

23. McKenna E, Traganos F, Zhao H, Darzynkiewicz Z. Persistent DNA damage caused by low levels of mitomycin C induces irreversible cell senescence. Cell Cycle. 2012;11(16):3132-40.

24. Mistry Y, Poolman T, Williams B, Herbert KE. A role for mitochondrial oxidants in stress-induced premature senescence of human vascular smooth muscle cells. Redox Biology. 2013;1(1):411-7.

25. Patel M, Day BJ. Metalloporphyrin class of therapeutic catalytic antioxidants. Trends in Pharmacological Science. 1999;20(9):359-64.

26. Zafarullah M, Li WQ, Sylvester J, Ahmad M. Molecular mechanisms of N-acetylcysteine actions. Cellular and Molecular Life Sciences. 2003;60(1):6-20.

27. Ahn JY, Park S, Yun YS, Song JY. Identification of a Novel and Potent Nrf2 inhibitor as a Radiosensitizer with High Throughput Screening. Transactions of the Korean Nuclear Society. 2009.

28. Pashin YV, Bakhitova LM, Bentkhen TI. Experimental study of the antimutagenic properties of 5-methylresorcinol. Bulletin of Experimental Biology and Medicine. 1985;100(1):912-4.

29. Jaworska A, Stojcevic-lemic N, Nias AHW, Sies H. The Effect of Paraquat on the Radiosensitivity of Melanoma Cells: the Role of Superoxide Dismutase & Catalase. Free Radical Research. 1993;18(3):139-45.

30. Kobori M, Shinmoto H, Tsushida T, Shinohara K. Phloretin-induced apoptosis in B16 melanoma 4A5 cells by inhibition of glucose transmembrane transport. Cancer Letters. 1997;119(2):207-12.

31. Sarbassov DD, Ali SM, Sengupta S, Sheen J-H, Hsu PP, Bagley AF, et al. Prolonged Rapamycin Treatment Inhibits mTORC2 Assembly and Akt/PKB. Molecular Cell. 2006;22(2):159-68.

32. Huang C, Ma W-y, Goranson A, Dong Z. Resveratrol suppresses cell transformation and induces apoptosis through a p53-dependent pathway. Carcinogenesis. 1999;20(2):237-42.

33. Yarrow JC, Totsukawa G, Charras GT, Mitchison TJ. Screening for Cell Migration Inhibitors via Automated Microscopy Reveals a Rho-Kinase Inhibitor. Chemistry & Biology. 2005;12(3):385-95.

34. Kessler M, Ubeaud G, Jung L. Anti‐and pro‐oxidant activity of rutin and quercetin derivatives. Journal of Pharmacy and Pharmacology. 2003;55(1):131-42.

35. Mitchell JB, Samuni A, Krishna MC, DeGraff WG, Ahn MS, Samuni U, et al. Biologically active metal-independent superoxide dismutase mimics. Biochemistry. 1990;29(11):2802-7.

36. Forrest VJ, Kang Y-H, McClain DE, Robinson DH, Ramakrishnan N. Oxidative stress-induced apoptosis prevented by Trolox. Free Radical Biology and Medicine. 1994;16(6):675-84.
